# Challenges for palliative care in times of COVID-19: a scoping review

**DOI:** 10.3389/fpubh.2024.1330370

**Published:** 2024-03-26

**Authors:** Marisa Lourenço, Tânia Gomes, Fátima Araujo, Filipa Ventura, Rosa Silva

**Affiliations:** ^1^Nursing School of Porto, Center for Health Technology and Services Research (CINTESIS), Porto, Portugal; ^2^Urology Department - Santo António University Hospital Center, Porto, Portugal; ^3^The Health Sciences Research Unit: Nursing (UICISA: E), Nursing School of Coimbra, Coimbra, Portugal; ^4^Centre for Evidence Based Practice: A JBI Centre of Excellence (PCEBP), Coimbra, Portugal

**Keywords:** palliative care, end-of-life care, COVID-19, health personnel, family

## Abstract

**Introduction:**

Many of the essential practices in palliative care (PC) had to be adapted to the COVID-19 pandemic. This global spread of the infectious respiratory disease, caused by SARS-CoV-2, created unprecedented obstacles. The aim of this research was to comprehensively assess the experiences and perceptions of healthcare professionals, individuals, and families in palliative and end-of-life situations during the COVID-19 pandemic.

**Methods:**

A scoping review was conducted using the databases CINAHL Complete, MEDLINE, Scopus, SciELO, Cochrane Central Register of Controlled Trials, Psychology and Behavioral Sciences, MEDIClatina, and Portugal’s Open Access Scientific Repository. The review followed the JBI^®^ methodological approach for scoping reviews.

**Results:**

Out of the initially identified 999 articles, 22 studies were included for analysis. The deprivation of relationships due to the safety protocols required to control the spread of COVID-19 was a universally perceived experience by healthcare professionals, individuals in PC, and their families. Social isolation, with significant psychological impact, including depersonalization and despair, was among the most frequently reported experiences by individuals in palliative situation. Despite healthcare professionals’ efforts to mitigate the lack of relationships, the families of these individuals emphasized the irreplaceability of in-person bedside contact.

**Systematic review registration:**

https://osf.io/xmpf2/.

## 1 Introduction

Advancements in science and technology have contributed to an augmentation in life expectancy and the management of numerous severe pathologies, yielding substantial enhancements in the quality of life. Nevertheless, this extension of lifespan poses the challenge of contending with progressive and advanced chronic illnesses, frequently precipitating frailty and reliance on assistance for daily activities. Within this framework, palliative care (PC) assumes an exceedingly critical role, furnishing assistance not solely on a physical plane, but also addressing emotional and spiritual needs, thereby assisting in the preservation of dignity and quality of life until the final moments. The terminal phase of existence is characterized by the encounter with myriad losses across pivotal spheres of the human condition, spanning physical, psychological, social, and spiritual dimensions. This revelation further underscores the indispensability of PC as a requisite response to the intricacy and varied needs encountered by individuals at this juncture of life (1, 3).

PC services are comprehensive and holistic, delivered by specialized teams to individuals of all ages experiencing suffering due to incurable or severe illness, in advanced and progressive stages, as well as to their families. These services are offered across various healthcare settings. The primary objective of PC is to enhance well-being and quality of life by preventing and alleviating physical, psychological, social, and spiritual distress (4). A person in an end-of-life situation is considered to have an estimated prognosis of 12 months, while a person in a terminal phase is typically considered as having an expected prognosis of 3–6 months (4).

PC is a demanding form of care that combines science and humanism. It emphasizes early identification of the person’s needs, rigorous control of suffering and distress, and the promotion of autonomy, up to the last days of life. PC advocates for quality of life and dignity throughout the process of dying and in death (5). It is a global imperative for health and equity and is universally regarded as a basic human right (6). PC chooses active interventions in various dimensions of suffering to prevent it from becoming disruptive for the person in extreme end-of-life circumstances. It strives for person-centered care and support to the family, aiming to enhance life, optimize human well-being, and maximize the dignity of care (7).

PC practitioners establish their actions upon a model of technical and humanized approach to care, which is necessarily personalized and primarily rooted in four fundamental pillars: (i) stringent control of symptoms, employing both pharmacological and non-pharmacological measures; and (ii) appropriate communication with the individual and their family, utilizing active listening strategies, approaches to promote dignity, and assisting in finding meaning for the remaining life, with emphasis on the significance of non-verbal communication (i.e., gaze, touch, facial expression, and hand placement); (iii) supporting the family, identifying their needs, mobilizing their potential, and aiding them in coping with the various losses before and after the patient’s death; (iv) interdisciplinary teamwork, integrating the contributions of different professionals adequately trained to address the diverse needs of the individual and their family (8).

On March 11, 2020, the World Health Organization (WHO) declared the status of a global pandemic due to the spread of COVID-19, a respiratory infectious disease caused by the SARS-CoV-2 virus (9). The global population faced intense and unprecedented challenges, leading to economic shifts, humanitarian crises, and new social interaction norms (10). The pandemic brought about abrupt and stringent changes in people’s lives, resulting in significant personal, psychological, professional, social, and familial impacts within a timeframe that, even today, remains poorly defined (11).

The overall circumstances experienced during 2020–2022 due to the COVID-19 pandemic, demanded a reevaluation of caring procedures, where a priority measure emerged: restricting contacts between individuals at the end of life, their families, and healthcare professionals. The need to prevent transmission both during hospitalization and at the home setting, significantly changed many aspects PC, especially concerning the doctor/professional/patient relationship (12). The use of masks and other personal protective equipment (PPE) substantially changed both verbal and non-verbal communication. The imposed physical distancing limited the possibility of providing a warm embrace or offering a comforting gesture (13). Consequently, facial expressions were also restricted, making the transmission of messages more challenging. Genuine and comprehensible communication relies heavily on facial expressions (14). Individuals in need of PC experienced long periods of solitude without the presence of their family members. Despite the fear of transmission and imposed isolation, these individuals managed to preserve their ability to communicate and interact, nearly until the moment of death (15). Since communication is an essential resource for high-quality PC, limitations in personal interaction among the individual, family, and healthcare providers significantly impacted the delivery of these services (15).

The information concerning the experiences perceived by healthcare professionals, individuals, and families regarding PC during the COVID-19 pandemic can provide significant insights about supporting strategies for PC practices in future pandemics. This information may also prove valuable during situations where healthcare decisions need to be made promptly.

This study aimed to map the experiences of healthcare professionals, individuals, and families in PC and end-of-life situations during the COVID-19 pandemic. An initial search in the JBI Evidence Synthesis, MEDLINE (PubMed), and CINAHL databases did not yield any scoping reviews addressing this objective.

The research questions guiding this study were as follows: (i) What are the experiences perceived by healthcare professionals in the practice of PC and end-of-life situations during the COVID-19 pandemic? (ii) What are the experiences perceived by the person in palliative or end-of-life situations during the provision of care in the context of the COVID-19 pandemic? (iii) What are the experiences perceived by the family of the person in palliative or end-of-life situations during the provision of care in the context of the COVID-19 pandemic? (iv) What strategies were employed by healthcare professionals during the COVID-19 pandemic in the practice of PC and end-of-life situations?

## 2 Method

The study followed the JBI® methodology for scoping reviews, which was considered the most appropriate method to explore and examine the experiences of healthcare professionals, individuals, and families in PC and end-of-life situations during the COVID-19 pandemic. Eligibility criteria were developed following the mnemonic P (participants), C (concept), and C (context) and with no deviations from the review protocol (16), with registration on the Open Science Framework.^1^

### 2.1 Eligibility criteria

Participants: studies that involved healthcare professionals from multidisciplinary teams providing PC and end-of-life care; individuals aged 18 and above and/or their families, who were in palliative and end-of-life situations, referred for PC.

Concept: studies that focused on the concept of experience and perception as encountered by individuals, families, and healthcare professionals regarding the provision of PC and end-of-life care during the COVID-19 pandemic. This also included studies referencing the strategies employed by healthcare professionals during the COVID-19 pandemic in the practice of PC and end-of-life care.

Regarding the Context, studies conducted in locations where PC and end-of-life care are provided were included. Studies published in English between 2019 and 2022 were incorporated, encompassing original research with various methodological approaches such as qualitative, quantitative, and mixed methods, as well as literature reviews, as outlined in the review protocol (16).

Literature that did not report on the SARS-COV-2 Pandemic period was excluded.

### 2.2 Search strategy

The research employed a comprehensive strategy to identify available literature regarding the experiences of healthcare professionals, individuals, and families in PC and end-of-life situations during the COVID-19 pandemic. An initial evidence search was conducted by one reviewer to determine key terms and develop the search strategy. This search was limited to MEDLINE (via PubMed) and CINAHL (via EBSCO). The search strategy was pilot tested in a database selected by the research team to ensure its robustness in capturing the necessary evidence before finalization. Text words found in the titles and abstracts of relevant articles, as well as indexing terms used to describe the articles, were used to develop a comprehensive search strategy in databases such as CINAHL Complete (via EBSCOhost), MEDLINE (via PubMed), Psychology, Mediclatina, and Cochrane Database of Systematic Reviews (via EBSCOhost). Grey literature was searched for in RCAAP (Repositório Científico de Acesso Aberto de Portugal). The search strategy incorporated all identified keywords and indexing terms, and the Boolean phrase was adapted for each included database. For additional information, please refer to Lourenço et al. (14). Reference lists of all selected studies were screened for additional relevant studies. The search strategy was developed by two researchers (17).

### 2.3 Study selection

**Figure 1:**
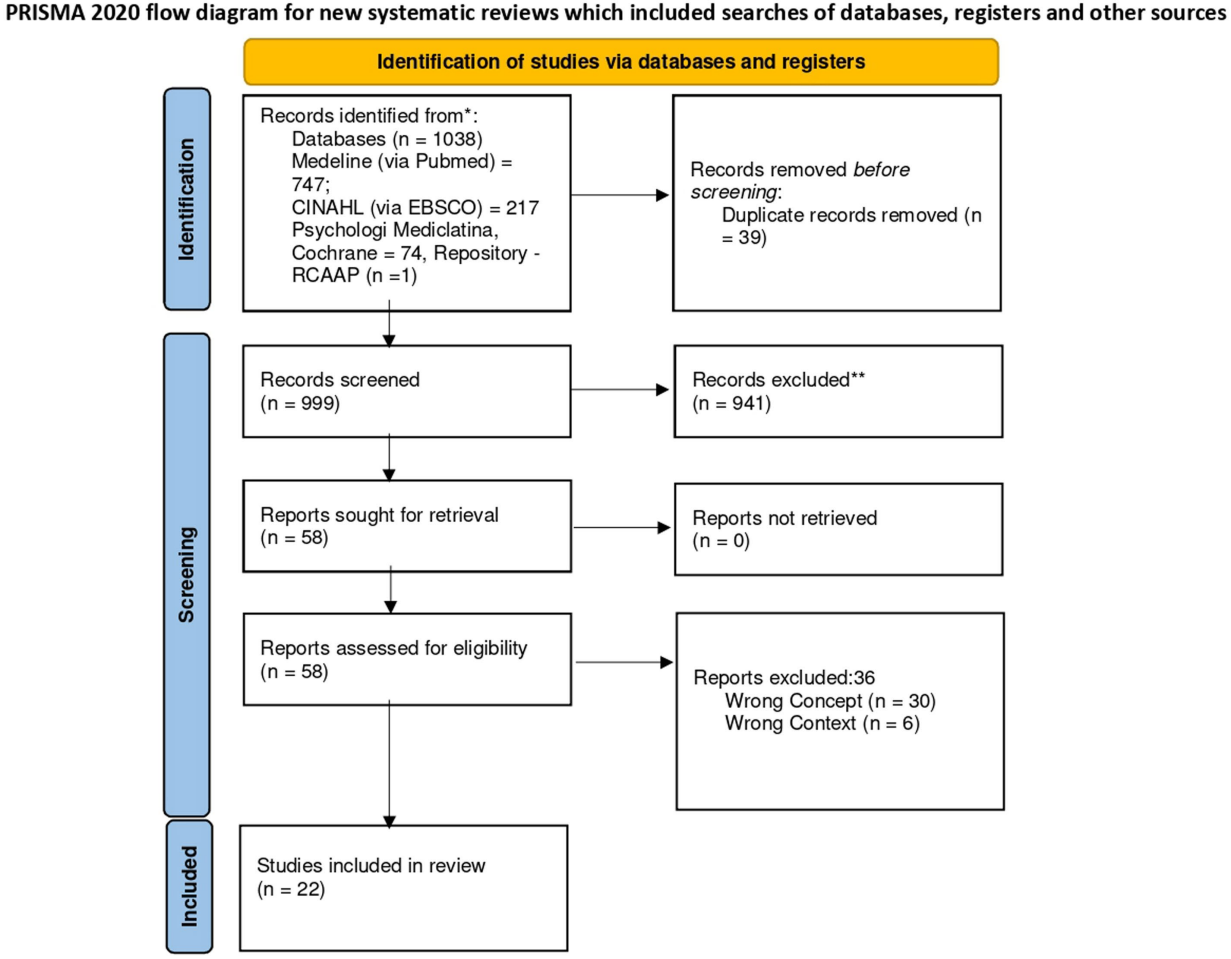
Search results and study selection and inclusion process (18).

The identified studies were retrieved and stored in Mendeley® V1.19.8 (Mendeley Ltd., Elsevier, Netherlands), and the duplicates were removed. Subsequently, the articles were imported into Rayyan QCRI (Qatar Computing Research Institute [Data Analytics], Doha, Qatar). Titles and abstracts were independently reviewed by two researchers to assess eligibility. The full texts of eligible documents were thoroughly examined by two independent reviewers based on the inclusion and exclusion criteria. Exclusion reasons for full-text articles that are documented in Figure 1. Discrepancies that emerged between reviewers during this stage of the selection process were solved through discussion with a third reviewer. The results of the search and the study inclusion process are reported following the Preferred Reporting Items for Systematic Reviews and Meta-Analyses (PRISMA) flow diagram (18).

### 2.4 Data extraction

Data were extracted from articles included in the scoping review by two independent reviewers using a data extraction tool developed by the reviewers and refined following piloting with a small number of studies, and subsequently applied to all included studies. The data extraction tool collected specific information about the population, context, geographical location, study methods, phenomena of interest relevant to the review objectives, and the type of sources. Discrepancies between the two reviewers were resolved through discussion or by involving a third reviewer. A reflective thematic analysis was conducted to extract findings related to common themes. The extracted data are presented in both tabular and diagrammatic formats. A narrative summary follows the tabulated results, according to JBI® recommendations (19), which align with the protocol planned for this study (16).

## 3 Results

### 3.1 Study inclusion

The research strategy yielded a total of 1,038 references, among which 39 were removed due to duplication, resulting in 999 articles for title and abstract screening. A total of 941 references were excluded during the title and abstract screening stage with a sample of 58 studies proceeding to full-text review. A sample of 22 references met the inclusion criteria, being the research sample of this scoping review. Figure 1 provides a concise overview of the article screening process for inclusion in the review, following the PRISMA flowchart (18).

### 3.2 Characteristics of included studies/reports

The characteristics of the studies included in this review are presented in Table 1. Among the 22 studies included in this scoping review, 21 were primary studies (2, 20–29, 31–39), and one was a narrative literature review (30). The studies originated from various countries: 9 from the USA, 3 from Germany, 3 from Italy, 2 from the UK, 1 from Australia, 1 from Brazil, 1 from Canada, 1 from Ireland, and 1 from Portugal. The majority (*n* = 12) employed qualitative approaches (2, 20, 22, 26–28, 33, 35–39), while the remaining ten were quantitative studies (21, 23–25, 29–32, 34, 37). Regarding study types, 15 were observational studies, of which 13 were descriptive studies (2, 21, 23, 24, 29, 31–39), and two were cross-sectional designs (25, 37). The remaining studies included three case studies (20, 22, 26), one narrative literature review (30), and three qualitative studies (27, 28, 39). These qualitative studies aimed to comprehend the grieving process (27), the changes and challenges in the daily work of healthcare professionals (39), and the impact of changes on individuals and caregivers in palliative and end-of-life situations (28), exploring the experiences of participants including bereaved family members, professionals, and patients/caregivers throughout the study duration.

**Table 1:** Study characteristics concerning the country of origin, study design, number of participants, types of experiences perceived and described in the study, and care strategies.

Study	Study design, Context and Sample (*N*)	Aim	Focus of the perceived experiences			Care strategy	Country
			Healthcare professionals	The person	The family		
Delisle et al. (18)	Case Study: Older adult individual (hypertension and early-stage dementia; hypoxic respiratory failure due to COVID-19 pneumonia); transferred from the intensive care unit to the PC unit.	Illustrate the importance of PC in the favorable outcome of an older adult individual with COVID-19 pneumonia, undergoing an extended period of failed attempts at discontinuation of mechanical ventilation in the intensive care setting.	x	x	x	_	New York
Samara et al. (20)	Descriptive Study; 28 nursing homes; Data collected over 3 months during 193 family conferences.	Adapt and test the "Needs Rounds" model using telehealth in the context of the COVID-19 pandemic.	x	_	_	x	Australia
Tanzi et al. (21)	Holistic case study using data triangulation. Nine physicians and 22 nurses from the Infectious Diseases Unit participated, along with two doctors from the PC Unit (inter-hospital PC team).	Describe the interventions carried out by the PC team in response to the intervention needs of the professionals from an infectious diseases unit during the COVID-19 pandemic. Identify necessary changes to enhance care, considering the COVID-19 outbreak in a hospital setting.	x	_	_	x	Italy
Broglio & Kirkland (22)	Descriptive study (quantitative approach, survey querying the advantages/disadvantages of PC consultations via telemedicine). *N* = 199 PC patients in a rural context.	Examine the perception of a group of individuals in palliative situations regarding telemedicine medical consultations during a recommended period of social isolation due to the COVID-19 pandemic.	x	x	_	_	USA
Meyerson et al. (23)	Retrospective study of the cases of individuals who died due to COVID-19 complications in the Intensive Care Unit; Medical comorbidities included cardiovascular and pulmonary diseases, diabetes mellitus, obesity, hematological disorders, active malignancies, and neurological disorders, including dementia. *N* = 33.	Identify factors for caring for an older adult population with COVID-19. Provide guidelines for teams caring for older adult individuals with medical comorbidities and COVID-19.	x	_	_	x	USA
Trianti et al. (24)	Correlational-descriptive study (survey, quantitative approach). *N* = 154 participants from three distinct contexts: (i) inpatients in a PC unit; (ii) individuals receiving PC as outpatients; (iii) 65 individuals visiting a general clinic.	Identify the level of anxiety induced by COVID-19 in individuals in palliative situations across various care contexts. Compare the anxiety levels of these individuals with those visiting a general clinic. Identify the impact of COVID-19-induced anxiety on daily life.	_	x	_	_	Germany
Mercadante et al. (25)	Descriptive study (interviews - qualitative approach), 16 Family members of individuals hospitalized in the PC unit.	Determine the satisfaction level of family members of patients admitted to acute PC units and hospices regarding their participation in clinical visits/meetings through WhatsApp	_	_	x	x	Italy
Stockdill, et al., 2021 (26)	Case study (78-year-old African American woman with recurrent breast cancer, pulmonary metastasis, and dyspnea; Baptist, mother of 5, grandmother of 10, and great-grandmother of 3; receiving Palliative Chemotherapy); developed dyspnea; was intubated in the intensive care unit. Admitted 80 km away from her residence area (had no visitors due to distance and COVID-19 restrictions). Died from cardiac arrest, alone after a resuscitation attempt.	Present the current evidence and best practices in telehealth use in PC. Explore how telehealth can be utilized for the provision of early PC in a virtual format. llustrate how the process would be conducted in this scenario if strategies like ENABLE and Vital Talk were employed in addressing the patient and their family.	_	_	x	x	USA
Macchi et al. (27)	Qualitative study using semi-structured interviews, responses to open-ended questions, and analysis of clinical documentation - clinical processes. *N* = 108 individuals with Parkinson's disease, Alzheimer's disease, or related disorders; -90 caregivers enrolled in a community-based outpatient PC clinical trial	Generate a person-centered description, based on their perspectives, regarding the impact of COVID-19 on patients living with neurodegenerative diseases and caregivers.	x	x	x	x	USA
Gelfman et al. (28)	Descriptive study about the response of the Mount Sinai Health System (MSHS) during the month of April 2020, addressing the needs of end-of-life patients due to COVID-19. *N* = 1,019 patients received specialized PC consultations across MSHS inpatient facilities.	Describe the rapid expansion and establishment of new specialized PC services within the healthcare system to meet the needs of COVID-19 patients in New York City.	x	_	_	x	USA
Powell & Silveira (29)	Theoretical review article	Examine how PC teams can support older adult individuals with multimorbidity and distressed caregivers during the COVID-19 pandemic.	x	x	x	x	USA
Tielker et al. (30)	Quantitative study within the paradigm (online questionnaire using Unipark). The study is part of a collaborative German project: "National Strategy for PC for Severely Ill and End-of-Life Patients and Their Families during Pandemics (PallPan)." *N* = 410 clinicians.	Describe the experiences, challenges, and perspectives of general practitioners regarding end-of-life care during the first peak of the COVID-19 pandemic (spring 2020) in Germany.	x	_	_	_	Germany
Kamal et al. (31)	Mixed-method study (Case Report Form - CRF), focused on new experiences with COVID-19, comprising 13 items (12 with direct response options and 1 open-ended); The form was distributed across multiple social networks to specialized PC teams. A total of 306 records were submitted, created by different healthcare professionals (physicians, advanced practice nurses, social workers, and chaplains); out of these, 298 records were related to adults, and 8 were related to children or infants	Describe the real-time experiences of specialized PC professionals while caring for individuals with COVID-19.	x	_	_	_	USA
Schloesser et al. (32)	Descriptive-observational study (Online survey, incorporating closed and open-ended questions) The study is part of a collaborative German project titled "National Strategy for Palliative Care," led by the National Network of University Medicine Research, focusing on COVID-19. *N* = 81 bereaved family members.	Describe the experiences of bereaved family members of patients who died during the COVID-19 pandemic, regardless of whether they were infected or not.	_	_	x	_	Germany
Harasym et al. (33)	Qualitative study employing Michie's COM-B theoretical framework, which comprises three categories: Capability, Opportunity, and Motivation. *N* = 23 physicians, including 18 family physicians and 5 PC specialists. The study was conducted in long-term care facilities located in urban, rural, or both urban and rural settings (Northern, Edmonton, Central, Calgary, and Southern regions).	Explore barriers and facilitators of end-of-life support in frail adults, as well as the ideal support in long-term care facilities, through the experiences and perceptions of community and PC specialists who visit these units.	x	_	_	_	Canada
Feder et al. (34)	Descriptive study employing a qualitative approach. *N* = 324 families.	Examine the perception of bereaved families regarding the quality of end-of-life communication between older adult patients and staff in geriatric care centers in the USA during the COVID-19 pandemic.	_	_	x	_	USA
Kirby et al. (35)	Qualitative study using a semi-structured interview approach. N = 20 nurses providing care to individuals with suspected and confirmed oncological PC needs for COVID-19.	Identify the key influences in the care of individuals with oncological PC needs during the COVID-19 pandemic, as perceived by nurses.	x	_	_	_	Brazil
Hanna et al. (36)	Descriptive qualitative design employing semi-structured interviews. Participants included professionals providing end-of-life care in hospitals (*n* = 10), hospices (*n* = 3), and nursing homes (*n* = 3). The interviews were conducted via phone (*n* = 11) or Zoom (*n* = 5).	Explore the experiences and perceptions of healthcare professionals and social workers in providing end-of-life care during the COVID-19 pandemic in the United Kingdom. Examine these professionals' perceptions of the needs of families when a family member was dying during the COVID-19 pandemic. Understand the best support strategy for families with individuals dying during the COVID-19 pandemic.	x	_	_	x	United Kingdom
Franchini et al. (39)	Qualitative study conducted through semi-structured telephone interviews. The Grounded Theory framework was utilized in thematic analysis to identify key themes grounded in the interviews. Participants included 30 healthcare professionals (15 doctors and 15 nurses) providing home-based PC to cancer patients and their families within an Italian non-profit organization.	To comprehend the impact of the outbreak on home-based PC professionals, describing changes and challenges in their daily work, as well as their reactions to the COVID-19 pandemic	x	_	_	_	Italy
Mitchinson et al., 2021 (37)	A review of UK healthcare policies, semi-structured telephone interviews with healthcare professionals, and a review of written press articles were conducted. 28 interviews with healthcare professionals were included in the analysis.	Identify barriers to end-of-life care delivery, describe attempts at providing care during the COVID-19 pandemic, and understand the impact this had on healthcare professionals.	x	_	_	_	United Kingdom
Nestor et al. (37)	Cross-sectional study (qualitative survey approach) *N* = 250 healthcare professionals from a PC and older adult care unit.	Describe and characterize the magnitude and variety of ways in which the COVID-19 pandemic has affected the personal, social, and professional lives of healthcare professionals from a fully integrated PC and older adult care unit.	x	_	_	_	Ireland
Moreira (37)	Qualitative study within the qualitative paradigm (semi-structured interviews). *N* = 9 family members in the grieving process, ranging from one month to five months.	Understanding the grieving process of family members of patients in PC during the COVID-19 pandemic.	_	_	x	_	Portugal

PC, palliative care; UK, United Kingdom.

Among the 22 studies included in this review, 12 focus on PC in a hospital setting (20, 22, 24, 26, 27, 29, 30, 32, 35–38). Two studies highlight long-term care (21, 34), two explore PC in a home-based context (23, 39), and two investigate PC in outpatient settings (28, 31). In the remaining four studies (2, 25, 33, 37), the samples included participants from diverse contexts.

As shown in Table 1, most of the studies predominantly report the experiences and perceptions of healthcare professionals working in PC during COVID-19 (2, 20–24, 28–32, 34, 36, 37, 39). The experiences and perceptions of the person in palliative situation during COVID-19, were described in 5 studies (20, 23, 25, 28, 30). Concerning, the experiences and perceptions of the family of the person in palliative situations during COVID-19 these were reflected in eight studies (20, 26–28, 30, 33, 35, 38).

### 3.3 Review findings

#### 3.3.1 Theme 1: healthcare professionals’ experiences

Some of the experiences reported by healthcare professionals are described in Table 2. The theme of relationship and communication had the most significant impact. This care strategy was perceived differently by the PC team in relation to the person in a palliative and end-of-life situation (2, 22, 23, 30, 32, 37, 39) and the family (2, 21, 22, 28–32, 37, 39). The theme of relationship and communication was also evident between the multidisciplinary PC team and the frontline team caring for COVID-19 patients (22–24, 29, 30, 37, 39). Teams of professionals who provide PC have become managers in providing technical and instrumental support, in controlling complex symptoms, particularly in managing pain and dyspnea (2, 22, 24, 29, 30, 34, 39), in delicate end-of-life decisions and post-grief care (22, 24, 32), and in the emotional and psychosocial support provided to the frontline teams caring for COVID-19 patients (22, 24, 29, 30, 32). Other themes emerged in the practice of PC, such as procedures for caring for the person and the family in a palliative and end-of-life situation (31, 37, 39) and the impact on the well-being of the multidisciplinary PC team (2, 36, 37, 39) during the pandemic period, as presented in the data shown in Table 2.

**Table 2:** Perceived experiences of healthcare professionals in the context of palliative and end-of-life care during the COVID-19 pandemic.

Study	Perceived experiences of healthcare professionals
Delisle et al. (18)	The PC team focused on managing signs and symptoms, as well as addressing psychological and spiritual challenges. The team believed that this experience reinforced the belief that the holistic approach to patients and families, provided by the philosophy of PC, was truly life-affirming.
Samara et al. (20)	Family meetings and conferences conducted through telehealth enhanced access to specialized PC services, even during a pandemic. The implementation of telehealth could significantly expand access to specialized PC in rural and remote areas of Australia. Intervention through this model represented a cost-effective strategy for improving PC accessibility in nursing homes. Telehealth is as effective as in-person implementation.
Tanzi et al. (21)	The team focused on delivering difficult news over the phone to families and providing care to individuals in palliative situations. Comprehensive training was provided to the entire team in symptom management, particularly in controlling dyspnea, as well as in managing the suffering and death of patients. Involvement of a PC unit in intensive care can offer relief not only to the patients but also to healthcare professionals.
Broglio & Kirkland (22)	Physicians who used telemedicine found this experience very positive. They reported increased efficiency in documentation, scheduling, and pre/post-consultation processes. Telemedicine allowed for briefings with medical students and offered flexibility in scheduling. More consultations could be conducted via telemedicine compared to in-person visits. The percentage of cancelations and no-shows for telemedicine appointments decreased in comparison to in-person visits.
Meyerson et al. (23)	Physicians with PC training were frequently called upon to guide colleagues in the intensive care unit in making delicate end-of-life decisions. PC specialists were more involved in the care of most patients who died while hospitalized during the COVID-19 outbreak than intensivist physicians.
Macchi et al. (26)	Disruption in the delivery of healthcare and other support services. Limitation of telecommunications compared to in-person contact.
Gelfman et al. (28)	The teams had to restructure their routines to meet the needs of people with COVID-19 at the end of life, provide support in symptom control, and train communication skills. During the sessions, doctors empowered themselves to apply communication scripts tailored to communication needs, such as delivering news, and practiced applying these skills in care settings and virtually.
Powell & Silveira (29)	The teams consider that the most important PC actions during the COVID-19 pandemic period were communicating with people in palliative situations about their health condition, prognosis, hope, and fears, minimizing emotional suffering and reducing interpersonal conflict. PC providers assisted the sick individuals and their families in defining care goals. Older adult individuals, especially those with cognitive deficits, felt vulnerable without their family by their bedside.
Tielker et al. (30)	Approximately 61.5% of the respondents indicated that the quality of end-of-life care was maintained throughout the entire pandemic, while 36.8% held the opposite view. General practitioners conducting home visits for individuals with advanced chronic illness and at the end of life reported a 62.7% increase in phone contact and a decrease in in-person visits. 36.1% provided video consultations. 28.5% conducted fewer home visits, and 9.1% conducted more home visits compared to the pre-pandemic situation. Professionals noted that video conferences were used more by the family than by the person at the end of life. 48.5% of family members were prohibited from visiting the PC patient. Professionals perceived an exacerbation of the feelings of fear and loneliness in end-of-life patients in nursing homes. Professionals also observed increased distress among family members due to receiving less information from their ill relatives (85.9%) and the inability to support them with their physical presence (99.3%).
Kamal et al. (31)	The family was not allowed to visit the sick person due to the risk of COVID-19 contagion. Tense communication with the ill person and the family. Communication barriers between doctors and families. Rapid changes in medical management in PC. Difficulty in managing resources for post-discharge continuity of care. Challenges in conducting COVID-19 tests in outpatient settings. Guardianship and legal challenges. Delays in patient care due to slow judicial system response. Community resources are harder to obtain after discharge during this pandemic period. Increased emotional suffering due to lack of family visits. Challenge in visiting sick family members. Increased emotional distress, lack of perception about the quality of care, and hindrance in the family’s ability to understand the severity of their relative’s illness.
Harasym, et al. (33)	Involvement of the family in care. Lack of knowledge, family frailty, unrealistic expectations, and emotional reactions to grief and uncertainty were aspects considered essential by professionals for ideal family care. Capacity barriers include: lack of tools for symptom assessment, lack of knowledge and training in PC. Physical and social design barriers include: lack of dedicated spaces for death and mourning, inadequate professionals, and insufficient mental and spiritual health services for the population. Barriers and facilitators for community-based services. Most long-term care users would die at these centers, so these facilities should be designed to allow for death, dying, and grieving with dignity for these individuals and their families. Professionals were faced with a need for community resources. Support from psychology, church, and social work for individuals and families.
Kirby et al. (35)	Negative influences during care for individuals with advanced oncological illness in palliative situations during the COVID-19 pandemic: emotional burden related to symptoms that can trigger burnout syndrome, such as tachycardia (>100b/min), constant fatigue not alleviated by quality sleep, with physical and psychological repercussions. Anxiety, anguish, and irritability were cited as constant symptoms. Insecurity, negligence, and inattention when performing technical procedures. Fear and sadness related to the possibility of infecting their own family members, and when the team perceives the patient’s solitude at the end of life and the possibility of experiencing death alone. Positive influences: professional well-being during the pandemic due to required protective measures for professionals. Maintaining the same work team. Spirituality was considered by some participants as an important aid in the pandemic context, associated with the perception of connection and the significance of the caregiving process.
Hanna et al. (36)	Challenges and Facilitating Aspects in the Provision of End-of-Life Care during the COVID-19 Pandemic: Increased emotional demands; fatigue and exhaustion; fear of transmitting the disease to vulnerable people in their family circle, as well as the death of colleagues or relatives who contracted the virus; Providing end-of-life care in an unusual work context; Lack of preparation and confidence to provide psychological support to the individual and family in end-of-life situations; Anxiety when having to inform the family that the person was in the last days of life; End-of-life conversations with families held virtually; The absence of volunteers was also a loss of support for patients. Facilitators: professionals having access to clinical supervision or a grief counselor as part of the healthcare team; A vacation period was highly valued to promote the well-being of professionals; Creating a notification list to establish a set of principles in communicating imminent death; Increasing the number of phone calls to keep family members updated; Useful when families designated a ‘spokesperson’ to contact the healthcare team and provide updates on the patient’s clinical condition. Family Support Needs When the Patient Was Dying During the COVID-19 Pandemic: The needs of family members and the importance of providing support to the entire family in the last weeks/days of life were recognized. However, care provision was primarily focused on providing physical elements of care (symptom relief), with psychosocial support often limited to the family. Allowing a virtual relationship between the family and the person in the last weeks of life was very distressing. In the few situations where family members were able to spend time with the person in end-of-life, they provided some care. Final contact and saying goodbye to the person in end-of-life. Maintaining the bond between the individual and their family was crucial as death approached. “Navigating the grieving experience” was important for the family; however, deciding when to invite them for a visit created tensions within the healthcare teams. The professionals’ interpretation of “end-of-life” was not always unanimous. The family had to choose which member would visit the person in end-of-life, as only one person could make the visit, which was very difficult. The visit took place in the hospital reception area, interfering with the “private moment” between the family and the person in end-of-life. Occasionally, professionals reported providing activities for the person in end-of-life (playing favorite music, reading religious texts, spending more time with the person).
Franchini et al. (39)	Reorganization of Home PC during the Pandemic: Changes in daily routine, relationship, and communication with the patient and caregivers. Perception of the role of home PC professionals during the pandemic. Sense of responsibility; Importance of their role as home PC professionals; Home PC scenario in the local healthcare network during the pandemic. Participants’ perception of the critical role of home support in emergencies. Challenges in providing care within the local healthcare network; Centrality of home support services in the local healthcare network.
Mitchinson et al., 2021 (37)	Restriction of traditional care: policies related to care, increased work pressure, and a lack of human relationships limited the ability to provide quality end-of-life care and a peaceful death. Seeking new care approaches: These policies encouraged communication between individuals at the end of life and their families through video calls. Frustration arose when human relationships were not consistently prioritized. Healthcare professionals took personal actions in care to alleviate the suffering of the ill person and their family. When the family could not be present, they assumed the role of comforting those at the end of life. Establishment of identity and resilience: Healthcare professionals described challenges to their identity when care deviated from the quality standards they were accustomed to. Uncertainty often arose about whether what they were doing was right, especially when they were required to implement policies that contradicted their beliefs about end-of-life care. Prioritizing tasks that were meaningful to the individuals and their families helped the team accept difficult situations and improve feelings of self-efficacy. Adjusting their roles and striving to provide new forms of care seemed to empower the team and enable them to develop resilience.
Nestor et al., 2021 (37)	Most participants stated that their workload had changed significantly since the beginning of the pandemic and that their responsibilities were greater than before. They felt more stress and anxiety related to COVID-19, especially professionals in close proximity to the sick person. Almost two-thirds reported feeling the national support offered to frontline healthcare workers. Similarly, many felt adequately supported in their workplace. Few healthcare professionals reported significant changes in alcohol or tobacco consumption habits. There was stress and anxiety in both professional and personal contexts. The highest sources of stress were the fear of contracting COVID-19 or being a transmission vector to vulnerable and/or healthy family and friends. Having to care for and interact with sick, frail, or end-of-life individuals who were completely isolated. Having to implement constantly changing work protocols. Lack of social interaction with colleagues. Physicians, nurses, and healthcare assistants reported higher anxiety levels compared to other healthcare professionals. Healthcare professionals working exclusively in older adult care units reported higher stress levels compared to their counterparts specializing in other areas.

PC, palliative care.

#### 3.3.2 Theme 2: the person’s experiences

The perceptions experienced by individuals in palliative and end-of-life situations during the study period, due to the restrictions adopted by healthcare services associated with the risk of virus transmission, included: long periods of social isolation (20, 28, 30), which caused them negative psychological impact (20, 25, 28); and loss of dignity and insecurity (20) (for more details, see Table 3).

**Table 3:** Perceived experiences of the person in the context of palliative and end-of-life care during the COVID-19 pandemic.

Study	Perceived experiences of the person
Delisle et al. (18)	PC professionals promoted reorientation, recognition of personality, and reconnection with the most important people in their lives (children) by facilitating visits. PC provided more opportunities for social engagement. This supportive environment gave the ill person a sense of security and protection, promoting emotional well-being that should not be overlooked in the observed success in ventilator withdrawal.
Broglio & Kirkland (22)	Common reasons cited for continuing telemedicine include convenience (not having to leave home, drive, or find parking), cost savings (gas and transportation), and more personal attention. Those who preferred in-person meetings expressed concerns that non-verbal communication might not be captured over the phone/video, and that their emotional needs might not be met in telemedicine appointments. There was also a lack of trust in technology. It is noteworthy that this preference is related to clients who feel socially isolated, with in-person consultations being a potential opportunity for patient engagement with the PC team.
Macchi et al. (26)	The percentage of people reporting that anxiety caused by COVID-19 influenced their daily lives varied from 15 to 31% in the three groups, with no significant difference. The frequencies of insomnia caused by anxiety were low in all groups.
Powell & Silveira (29)	Increased symptomatic and psychosocial needs.

PC, palliative care.

#### 3.3.3 Theme 3: the family experiences

Regarding the experiences reported by the family, the relationship and communication with the person in a palliative situation are highlighted (20, 26, 27, 33, 38), as well as the need to stay by the bedside of the ill person despite all implemented strategies, such as telemedicine, to overcome some of the difficulties in relationship and communication (25, 26, 27, 29, 32, 43, 40). Additionally, there is a perceived sense of gratitude from the family toward the palliative care team (20). Experiences reported by families caring for the person in a palliative situation at home during COVID-19 reflect higher levels of burden (28, 30). However, one of the studies does not support this finding, as bereaved family members who accompanied their loved one at home expressed lower burden compared to those whose family member died in a hospital setting (33) (for more details, see Table 4).

**Table 4:** Perceived experiences of the person’s family in the context of palliative and end-of-life care during the COVID-19 pandemic.

Study	Perceived experiences of the person’s family
Delisle et al. (18)	The patient’s children witnessed a change in their father’s demeanor and prognosis. They reported that this experience allowed them to live out their religious faith, as well as express their gratitude to the care team.
Broglio & Kirkland (22)	Telemedicine proved to be very convenient (not having to leave home, drive, or find parking), cost-effective (saving on gas and transportation), and provided more personal attention. Those who preferred in-person meetings expressed concerns that non-verbal communication might not be captured over the phone/video and that their emotional needs might not be met in telemedicine appointments. There was also a lack of trust in technology.
Mercadante et al. (25)	The majority of family members viewed participation in virtual clinical visits favorably; however, most of them believed that in-person visits are irreplaceable.
Stockdill et al. (27)	The son who was informed about the outcome was disturbed by not being able to be physically close to his mother during her hospitalization, due to distance and pandemic-related restrictions. He was also distressed by not knowing how to communicate this bad news to his siblings and family.
Macchi et al. (26)	Increase in caregiver burden.
Powell & Silveira (29)	Family members felt guilty for leaving the person at end-of-life alone. The inability to stay with the ill person distances families from the most common means of receiving updates about the person’s clinical condition. Regular video conferences helped the person in a palliative situation and their families feel more connected to each other and to the medical team
Schloesser et al. (32)	Visitation Restrictions: Most bereaved family members felt overwhelmed by the visitation restrictions for the person at the end of life; some experienced pandemic-related stress; limitations were imposed on the duration or number of visitors. Place of Death: Relatives of individuals who died at home felt less burdened by the pandemic situation compared to when the person died in the hospital. Some family members reported that not working due to the pandemic allowed them to spend more time with the person at the end of life at home. Online Communication with the Person at End of Life: In the case of visitation restrictions, digital communication opportunities could serve as a way to connect with the family environment. The lack of equipment proved to be a hurdle. However, it was acknowledged that online communication could not compensate for physical contact. Communication with the Healthcare Team during the COVID-19 Pandemic: Most family members rated the emotional support from the healthcare team as good/excellent and felt treated with empathy after the death. However, around a dozen family members did not have contact with the healthcare team after their family member’s death.
Feder et al. (34)	Families identified contextual factors responsible for the quality of communication, including visitation restrictions, concern about the patient dying alone, and overall assessment of older adult care. Characteristics of high-quality communication included professionals’ availability to discuss the patient’s condition and care plan. On the other hand, low-quality communication was associated with limited access to the multidisciplinary team, lack of information about the patient’s condition, and when the family member was not consulted about healthcare decisions. The quality of communication with the person in a palliative situation was facilitated or hindered by the availability and use of remote technologies such as video.
Moreira (37)	Experience of the Disease Process / Perception of the Relative’s Health Status: Negative psychological experience (difficult, painful, threat of infection, anxiety, anticipation, restlessness, unrealistic view of the prognosis). Positive psychological experience (hope, tranquility, faith in God, realistic view of the prognosis). Positive everyday experience (regular visits and contacts, perception of in-person contact, ability to care, sufficient information, support from healthcare professionals, possibility of saying goodbye). Negative everyday experience (lack of information, inability to contact doctors, perception of no opportunity for goodbye, perception of information omission, pandemic-related restrictions). Psychological Impact of the News of Death (shock, anger, sense of unreality, nervousness, grief). Psychological impact post-loss (longing, sadness, desire to die, perception of the deceased’s presence, perception of lack of support). Facilitators of the grieving process (Psychological support, contact with the ill relative even if very brief, closeness to God, support from friends, informal social support, family support, psychological help, medical assistance, support from healthcare professionals, funeral rituals going as planned). Stressors/Challenges in the grieving process (Distancing from the ill relative, family conflicts, difficulty accepting reality, limitations due to the pandemic, financial problems, difficulties in previous bereavements). Coping strategies oriented toward action (returning to professional life). Coping strategies oriented toward emotional expression (expressing what I feel). Coping strategies oriented toward maintaining bonds (I talk to her as if she were beside me). Meanings attributed to the loss. Desire to continue with life. Desire to close the chapter, downplaying the pandemic. Enjoying life. Being at peace.

#### 3.3.4 Theme 4: strategies used by healthcare professionals

Despite the constraints resulting from the pandemic process, it was considered relevant to gather information about new strategies used by healthcare professionals in the practice of PC during COVID-19. Some studies addressed the use of new technologies to mitigate social isolation caused by the restrictive measures adopted by healthcare institutions (21, 22, 26, 28–30, 32, 38). Others discuss new strategies to ensure a peaceful death within the hospital (22, 30). Two of the studies addressed the strategies used to ensure the safety of the multidisciplinary team in infection control (29, 37) (see Table 5).

**Table 5:** Strategies used by healthcare professionals in the practice of palliative and end-of-life care during the COVID-19 pandemic.

Study	Perceived experiences of healthcare professionals
Samara et al. (20)	The previously validated “Needs Rounds” model, applied monthly in face-to-face settings, was tested on a weekly basis using telehealth.
Tanzi et al. (21)	Ensuring a peaceful death within the hospital. Discharge to home were prohibited to control the spread of COVID-19, even when requested by the person/family. Implementation of new strategies to support communication with families (e.g., phones and tablets). Implementation of hospital rules regarding the use of communication devices: communication devices had a new role in palliative care. Implementation of new strategies for delivering bad news.
Meyerson et al. (23)	Daily support from the PC team to the intensive care team, who cared for COVID-19 patients. The actions of these professionals were directed toward: the ill individuals during 24-h palliative care, daily direct contact with the patient and indirect contact with the family; they also provided training on symptom management, end-of-life care, and post-bereavement care.
Mercadante et al. (25)	Due to the restrictions imposed during the pandemic phase of COVID-19 (first wave), the palliative care team temporarily implemented the use of WhatsApp. The objective was to allow family members to participate in clinical visits, facilitating the exchange of information about the clinical progress and sharing decisions.
Stockdill et al. (41)	Recommendation to adapt the ENABLE and Vital Talk methods to address PC in individuals with oncological diseases and improve the relationship with healthcare professionals, providing skills training through telehealth. They advocate for maintaining this virtual strategy even after the pandemic to offer early PC in home settings, especially in rural and remote populations, as the majority of palliative care centers are located in major urban clinical centers.
Macchi et al. (26)	Telemedicine helped improve access to healthcare.
Gelfman et al. (28)	Structural adaptation of the outpatient PC unit into in-patient PC units and the adaptation of care: specialized symptom management and end-of-life care for individuals with COVID-19. Wards were converted to negative pressure and modified for continuous monitoring of the ill person. Measures were taken to minimize the team’s exposure to potential infection. For example, extension of gas lines for ventilators placed outside rooms, installation of transparent glass panels on all doors, high-resolution cameras for video feeding, connected to the nursing station. Specialized intravenous lines for infusion pumps were installed, located outside the rooms. Intensive care units included specialized palliative care nurses, postgraduate fellows, and/or assistant physicians in palliative care on their teams. These professionals primarily focused on identifying individuals with complex symptoms and addressing their symptom management needs.
Powell & Silveira (29)	PC teams opened their practices to patients and caregivers through phone and video, and they were quick to issue guidelines and recommendations for the best practices in telemedicine. The PC teams became the connecting link and advocates for families who could not visit hospitalized patients, providing daily updates to families. They also helped facilitate regular video conferences between the family and the patient, even when the person was unconscious.
Hanna et al. (36)	Tools such as quick question lists and daily family communication charts could help promote informative engagement of the family during restricted visitation times. Clear guidance and management of the appropriate timing for family visits to the end-of-life individual in institutional settings during a pandemic are necessary. Recommendations should be explicit, and contact should be facilitated when death is expected in weeks and days rather than hour(s) before death. There is a need for leadership and visible support within healthcare teams to promote self-care and reflection, as well as continuous access to psychological support for healthcare professionals and social workers.

PC, palliative care.

## 4 Discussion

Regarding the characterization of the studies included in this review, a wide geographical distribution was observed, covering various continents, although with a predominance of European, *n* = 10 (Germany, Italy, UK, Ireland, and Portugal), and American (*n* = 9) studies. The worldwide spread of included studies, reflect the global spread of the disease across different continents which is aligns with the concept of pandemic attributed to COVID-19. In this sample of 22 studies, there was a predominance of representation of PC in the hospital setting compared to home and long-term care settings. This result can be explained by the fact that most of the studies collected data in 2020 (during the first and second waves of the pandemic) due to fear of the unknown, the high rate of hospitalization among the older adult population with a high burden of disease and frailty.

To address the guiding questions of the review, the discussion was organized by exploring the experiences/perceptions of each participant group: healthcare professionals, individuals, and families in palliative and end-of-life situations. The analyzed studies revealed that communication was a universally perceived experience among the participants. This perception emphasizes the idea that appropriate communication with the individual and the family is a fundamental pillar of PC. In other words, communication among healthcare professionals, the individual, and the family should be systematic, centered on promoting dignity, and helping find meaning in the remaining life (40). The lack of relationship and communication associated with the protective measures required to control COVID-19 transmission was felt by individuals in palliative and end-of-life situations, especially due to social isolation and separation from loved ones. Additionally, the use of personal protective equipment impacted communication and touch (20). To enhance the comfort of the individual and the family, healthcare professionals adopted new forms of communication through technologies such as video calls. However, they felt frustrated when human connections were not consistently prioritized (18). The various participants valued new technologies, although professionals placed greater importance on them compared to families. The latter group considered these technologies an alternative strategy to alleviate the feeling of separation, emphasizing that they do not substitute for physical presence.

Concerning the experiences/perceptions of healthcare professionals in PC during COVID-19, the findings highlighted the following themes: the PC team acting as managers in providing technical and instrumental support, especially in the management of complex symptoms, particularly pain and dyspnea, in delicate end-of-life decisions, and in emotional and psychosocial support for frontline teams caring for individuals with COVID-19. These teams are familiar with legal requirements for documenting advance care directives, as well as living wills and decisions regarding appropriate life-sustaining measures. PC teams made efforts to maintain a connection with families who could not visit their hospitalized loved ones and provided post-bereavement care. They sought to ensure that the wishes of individuals in palliative situations and their families were properly documented and communicated.

Some changes in the procedures for caring for individuals and families in palliative and end-of-life situations, implemented by healthcare management entities, significantly increased the workload and responsibilities compared to the pre-COVID-19 period, conflicting with the principles of palliative care. These factors had an impact on the well-being of the multidisciplinary team (36, 39). Conversely, the well-being of healthcare professionals was observed when they used the required personal protective measures and maintained the same team. Some participants regarded spirituality as a binding factor in the caregiving process (36).

The experiences/perceptions of individuals in PC and at the end of life during COVID-19 received less attention in scientific literature. Among the five articles in which the palliative individual participated, in addition to communication, the following themes were identified: social isolation, psychological impact, loss of dignity, and insecurity. This was experienced due to the inherent restrictions during the pandemic period, which overlapped with the core principles of PC that truly affirm life. The overwhelming majority of studies reported the social isolation of patients, which had a significant psychological impact, including feelings of depersonalization and despair.

The psychological impact of the COVID-19 pandemic on individuals in PC admitted to PC units seemed insignificant, given the imminent threat that terminal illness represents for these individuals, in contrast to individuals receiving outpatient palliative care, who exhibited higher levels of anxiety (25). COVID-19 added a new dimension of suffering to the experience of individuals transitioning to palliative and end-of-life situations due to the serious and irreversible nature of the disease. These mostly older individuals found themselves socially isolated for an extended period. They were cared for by healthcare professionals whose presence was obscured by layers of personal protective equipment, disruption of circadian rhythms, and within an intensive care context, despite their palliative condition. These aspects led to an end-of-life process marked by insecurity and a lack of dignity (20). This contradicts the principles in healthcare disciplines where dignity is considered a professional code and a care policy (20). The findings confirm that institutions that allowed palliative care teams to assume full responsibility for managing individuals at the end of life facilitated a more dignified dying process (31).

The experiences/perceptions of the family of individuals in PC and at the end of life during COVID-19 revealed two central themes: staying by the bedside and the burden of concerns. Indeed, family members valued virtual contacts with the healthcare team and their loved ones, both in virtual clinical visits to monitor the situation (38) and to feel more engaged (20, 30, 35). These contacts were also helpful in receiving emotional support from the PC team during the illness and post-death period (27, 33). Therefore, family members express a sense of gratitude toward the PC team (20). However, they emphasize that in-person contact by the bedside of the family member is irreplaceable (38, 40). Communication enables a connection between different elements, facilitating shared decision-making (41).

Caregivers of individuals in palliative care and at the end of life perceived an increased burden during the COVID-19 period (28, 30), especially during the first and second waves of the disease. The abrupt constraints and restrictions, fear, and insecurity about the unknown may justify the heightened perception of burden among family members, regardless of whether the individual in palliative care and at the end of life was in a home or hospital setting.

The results indicate an increased burden on family caregivers providing care to individuals in palliative care and at the end of life in a home setting. The lack of psychosocial support reduced home-based PC, worsening condition of their family member, and imposed changes for pandemic control might explain this intensified perception of burden (30). However, when comparing the burden between caregivers providing care at home and those for who the loved one passed away at a hospital setting, the findings revealed that the former experienced less burden by the pandemic situation compared to the latter. According to these family members, not being employed due to the pandemic allowed them to spend more time with their family member at the end of life (33).

The new strategies used by healthcare professionals in the practice of PC during COVID-19 include the use of information and communication technologies to address communication and care documentation deficits. There was a reorganization of services and restructuring of CP units to cope with the rapid and exponential increase in COVID-19 infected individuals. Information technologies used to meet the universal need for social isolation had two levels of acceptance. The convenience of not having to leave home, drive or park a car, cost reduction (gas and transportation), and the perception of personalized care were seen as advantages. On the other hand, those who preferred in-person meetings expressed concerns that non-verbal communication might not be captured in phone/video consultations, emotional needs might not be met in telemedicine appointments, and there was a general mistrust on technology. It is worth noting that this preference was related to individuals who experienced social isolation, with in-person consultations seen as a potential opportunity for patient engagement with the PC team (23).

Visitor restrictions were a practice implemented by healthcare institutions worldwide to mitigate the pandemic. PC teams became the link and advocates for families who could not visit their hospitalized loved ones. They facilitated and promoted regular video conferences between families and patients (even when the person was unconscious) and provided daily updates to families about the clinical status of their loved ones, significantly reducing levels of distress (30).

The reorganization of services and restructuring of PC units in response to a public health emergency caused by the COVID-19 pandemic forced PC teams to make rapid decisions and adapt care to the new needs of individuals and families. As a result, many of the golden rules of PC practice had to be adjusted to the current COVID-19 pandemic. Coordination of care could be improved when the whole healthcare team was involved (22).

### 4.1 Limitations

The high proportion of descriptive studies and case studies, which are methodologically weak and less robust in terms of the evidence produced, requires caution in interpreting the results of this review.

## 5 Conclusion

The pandemic caused by COVID-19 imposed implications on healthcare professionals, individuals, and families in PC and end-of-life situations. Apart from the risk of contracting the disease, there was a need to adopt isolation policies that ended up changing many PC and end-of-life practices. The fear of virus transmission and the isolation imposed by health regulatory authorities led the participants to new experiences. The central theme reported by the participants during the study period was relationships. This caregiving strategy was perceived differently by all participants. The restriction of visits was a practice implemented by healthcare institutions worldwide, aiming to control and mitigate the pandemic disease. However, PC teams became the link and advocates for families who could not visit hospitalized patients. They promoted and facilitated regular videoconferences between the family and the patient. Nevertheless, individuals and families emphasized that despite the benefits of new technologies, in PC, in-person contact by the bedside was irreplaceable. The restrictive measures inherent to the pandemic period conditioned feelings of social isolation, with negative psychological impact, loss of dignity, and insecurity, especially for the person in PC. These factors overshadowed the fundamentals of PC.

## Data availability statement

The original contributions presented in the study are included in the article/supplementary material, further inquiries can be directed to the corresponding author.

## Author contributions

ML: Formal analysis, Writing – review & editing, Writing – original draft, Validation, Supervision, Project administration, Methodology, Investigation, Conceptualization. TG: Writing – original draft, Investigation, Conceptualization. FA: Project administration, Writing – review & editing, Supervision, Methodology. FV: Writing – review & editing. RS: Writing – review & editing, Methodology.

## Footnotes

^1^ https://osf.io/xmpf/
